# Supplementary material for: The external validity of machine learning-based prediction scores from hematological parameters of COVID-19: A study using hospital records from Brazil, Italy, and Western Europe
Source: PLoS One. 2025 Feb 4;20(2):e0316467. doi: 10.1371/journal.pone.0316467 (PMC11793750; doi:10.1371/journal.pone.0316467)
Supplement: S1 Appendix — (DOCX) [file pone.0316467.s001.docx]

**S1 Appendix:** 5 supporting tables and additional description on the webserver development

S1 Table: Different types of features included in dataset 1

| Feature Type | Age data | Hospital admission status | Haematological parameters | Co-morbidities |
| --- | --- | --- | --- | --- |
| Feature Name(s) | Patient Age Quantile | Regular Ward  Intensive Care Unit  Semi-Intensive Care Unit | Monocytes  Hematocrit  Leukocytes  Eosinophils  Hemoglobin  Lymphocytes  Red Blood Cells  Mean Platelet Volume  Rbc Distribution Width  Mean Corpuscular Volume | Adenovirus  Influenza A  Influenza B  Parainfluenza 1  Parainfluenza 2  Parainfluenza 3  Parainfluenza 4  Coronavirus 229E  Metapneumovirus  Coronavirus NL63  Coronavirus HKU1  Coronavirus OC43  Bordetella Pertussis  Rhinovirus/Enterovirus  Influenza B (Rapid Test)  Influenza A (Rapid Test)  Influenza A (H1N1-2009)  Respiratory Syncytial Virus  Chlamydophila Pneumoniae |

S2 Table: Chi-squared values between imputed and non-imputed datapoints in a) dataset 2 and b) dataset 3.

a)

| **Features** | **p-Value** |
| --- | --- |
| Sex | 0 |
| Age | 0 |
| Serum-Calcium | 0 |
| CK(U/L) | 0 |
| Serum-Creatinine | 0 |
| ALP(U/L) | 0 |
| GGT(U/L) | 0 |
| Glucose | 0 |
| AST(U/L) | 0 |
| ALT(U/L) | 0 |
| LDH(U/L) | 0 |
| Urea | 0 |
| Leukocytes(billions/L) | 0 |
| Erythrocytes(trillions/L) | 0 |
| Hemoglobin | 0 |
| Hematocrit(%) | 0 |
| MCV | 0 |
| MCH | 0 |
| MCHC | 0 |
| Platelets(billions/L) | 0 |
| Neutrophils(%) | 0 |
| Lymphocytes(%) | 0 |
| Monocytes(%) | 0 |
| Eosinophils(%) | 0 |
| Basophils(%) | 0 |
| Neutrophils(billions/L) | 0 |
| Lymphocytes(billions/L) | 0 |
| Monocytes(billions/L) | 0 |
| Eosinophils(billions/L) | 0 |
| Basophils(billions/L) | 0 |
|  |  |
|  |  |

b)

| **Features** | **p-Value** |
| --- | --- |
| ALT(U/L) | 0 |
| AST(U/L) | 0 |
| Basophils(counts/µL) | 0 |
| Eosinophils(counts/µL) | 0 |
| Erythrocytes(millions/µL) | 0 |
| GGT(U/L) | 0 |
| Hematocrit(%) | 0 |
| LDH(U/L) | 0 |
| Leukocytes(counts/µL) | 0 |
| Lymphocytes(counts/µL) | 0 |
| Mature-Neutrophils(counts/µL) | **2.27E-245** |
| Monocytes(counts/µL) | 0 |
| Neutrophils(counts/µL) | 0 |
| pO2-Arterial(mmHg) | 0 |
| Serum-Albumin(g/dL) | 0 |
| Serum-Calcium(mmol/L) | 0 |
| Serum-Ferritin(ng/mL) | 0 |
| Serum-Magnesium(mEq/L) | 0 |
| Serum-Phosphorus(mg/dL) | 0 |
| Total-Bilirubin(mg/dL) | 0 |
| Platelets(thousands/µL) | 0 |

S3 Table: 10-fold cross-validation results. Average and standard deviation (within parenthesis) were shown.

| Dataset | Accuracy | Sensitivity | Specificity | AUC score |
| --- | --- | --- | --- | --- |
| 1-four-feature | 0.83(0.04) | 0.66(0.19) | 0.85(0.02) | 0.87(0.07) |
| 2-four-feature | 0.74(0.03) | 0.75(0.04) | 0.73(0.07) | 0.79(0.03) |
| 1-fourteen-feature | 0.86(0.04) | 0.60(0.16) | 0.90(0.04) | 0.87(0.04) |
| 3-fourteen feature | 0.74(0.01) | 0.72(0.04) | 0.75(0.01) | 0.81(0.02) |

S4 Table: Performance of different ML algorithms on all the four sub-datasets.

| Dataset | Model | Sensitivity | Specificity | Accuracy | AUC Score |
| --- | --- | --- | --- | --- | --- |
| Dataset | Model | Sensitivity | Specificity | Accuracy | AUC Score |
| 1-fourteen feature | XGBoost | 0.750 | 0.887 | 0.869 | 0.922 |
|  | Logistic regression | 0.375 | 0.981 | 0.902 | 0.915 |
|  | Naïve Bayes | 0.5 | 0.83 | 0.787 | 0.866 |
|  | Fisher Linear Discriminant | 0.125 | 0.981 | 0.869 | 0.920 |
|  | K-Nearest Neighbour | 0.25 | 0.943 | 0.852 | 0.818 |
|  | Random Forest | 0.125 | 0.981 | 0.869 | 0.844 |
|  | Support Vector Machine (SVM) | 0 | 1 | 0.869 | 0.891 |
| Dataset | Model | Sensitivity | Specificity | Accuracy | AUC Score |
| 1-four-feature | XGBoost | 1.000 | 0.906 | 0.918 | 0.939 |
|  | Logistic regression | 0.125 | 1 | 0.885 | 0.920 |
|  | Naïve Bayes | 0.750 | 0.868 | 0.852 | 0.882 |
|  | Fisher Linear Discriminant | 0.125 | 1 | 0.885 | 0.887 |
|  | K-Nearest Neighbour | 0.5 | 0.925 | 0.869 | 0.873 |
|  | Random Forest | 0.125 | 0.962 | 0.852 | 0.928 |
|  | Support Vector Machine (SVM) | 0 | 1 | 0.869 | 0.871 |
| Dataset | Model | Sensitivity | Specificity | Accuracy | AUC Score |
| Dataset | Model | Sensitivity | Specificity | Accuracy | AUC Score |
| 2-four-feature | XGBoost | 0.845 | 0.733 | 0.787 | 0.842 |
|  | Logistic regression | 0.786 | 0.789 | 0.787 | 0.815 |
|  | Naïve Bayes | 0.786 | 0.433 | 0.603 | 0.740 |
|  | Fisher Linear Discriminant | 0.762 | 0.733 | 0.747 | 0.800 |
|  | K-Nearest Neighbour | 0.738 | 0.744 | 0.741 | 0.776 |
|  | Random Forest | 0.631 | 0.689 | 0.661 | 0.765 |
|  | Support Vector Machine (SVM) | 0.821 | 0.756 | 0.787 | 0.830 |
| Dataset | Model | Sensitivity | Specificity | Accuracy | AUC Score |
| Dataset | Model | Sensitivity | Specificity | Accuracy | AUC Score |
| 3-fourteen-feature | XGBoost | 0.784 | 0.733 | 0.746 | 0.842 |
|  | Logistic regression | 0.252 | 0.945 | 0.773 | 0.774 |
|  | Naïve Bayes | 0.870 | 0.457 | 0.560 | 0.732 |
|  | Fisher Linear Discriminant | 0.007 | 0.989 | 0.747 | 0.742 |
|  | K-Nearest Neighbour | 0.422 | 0.901 | 0.782 | 0.747 |
|  | Random Forest | 0.339 | 0.931 | 0.784 | 0.785 |
|  | Support Ve0ctor Machine (SVM) | 0 | 1 | 0.751 | 0.742 |

S5 Table: elapsed computational time for different ML models (time in seconds)

| Dataset | XGBoost | Logistic Regression | Gaussian Naïve Bayes | Linear Discriminant | K Nearest Neighbour | Random Forest | Support Vector Machine |
| --- | --- | --- | --- | --- | --- | --- | --- |
| 1 | 1.68 | 0.27 | 0.007 | 0.09 | 0.002 | 0.20 | 38.02 |
| 1a | 0.15 | 0.02 | 0.001 | 0.005 | 0.0008 | 0.03 | 0.14 |
| 1b | 0.13 | 0.006 | 0.001 | 0.002 | 0.001 | 0.03 | 0.10 |
| 1c | 0.09 | 0.003 | 0.001 | 0.001 | 0.0008 | 0.02 | 0.01 |
| 2a | 0.68 | 0.023 | 0.001 | 0.013 | 0.007 | 0.10 | 0.71 |
| 2b | 2.76 | 0.004 | 0.001 | 0.003 | 0.001 | 0.04 | 0.35 |
| 3a | 1.66 | 0.087 | 0.004 | 0.04 | 0.003 | 0.27 | 9.73 |
| 3b | 2.41 | 0.067 | 0.007 | 0.05 | 0.06 | 0.36 | 26.65 |
| Model average | 1.19 | 0.06 | 0.003 | 0.026 | 0.01 | 0.13 | 9.47 |

**Design of the web server:**

The web server hosted two different models, a four-hematological parameter model and a fourteen-hematological parameter model. The web server was developed on an HTML framework, with five working HTML files: a landing page and two pages each for each method, one for data input and the other for prediction display. The basic skeleton of the HTML files was formatted with CSS code, and these files were deployed via the Python module, Flask. Python libraries, like numpy and pandas, were used to collect and process the input, with the responses generated by the XGBoost models.
