# Supplementary material for: The external validity of machine learning-based prediction scores from hematological parameters of COVID-19: A study using hospital records from Brazil, Italy, and Western Europe
Source: PLoS One. 2025 Feb 4;20(2):e0316467. doi: 10.1371/journal.pone.0316467 (PMC11793750; doi:10.1371/journal.pone.0316467)
Supplement: Attachment — Submitted filename: Response-to-the-reviewers.docx [file pone.0316467.s002.docx]

To,

The Editor,

PLOS ONE,

Subject: Rebuttal letter

Dear Sir,

Please find below the response to the reviewers (in red fonts). All the line numbers are mentioned according to the Word document, named “Manuscript_revised_18112023_all_markup”. We tried our best to answer all the points raised by the reviewers. We are confident that the incorporation of valuable suggestions from the esteemed reviewers has improved the quality of the manuscript.

Looking forward to your positive response.

Yours sincerely,

Debashree Bandyopadhyay

Rebuttal:

Reviewer #1: The following points should be considered in the manuscript:
1-The authors have focused on specific type of the haematological parameters without adding any explanations about the reasons of the using. So, the authors must explain this significant point in details.

Response: The explanation for choosing specific hematological parameters were added in “Results and Discussion” section, subheading – “*Correlation between features and SARS-COV-2 results in different clinical datasets”, line number (version with all-markup):370-442 and 453-468.*

2-The authors must explain in more and more details how they got the actual positive results of applying the model which they focused on in the manuscript on the biological tissues effects.

Response: To the best of our knowledge, biological tissue effects were not studied in this work.

Here we suppose “actual positive results” indicate the “ground truth” used in this study for comparison with the predicted results. The “actual positive results” were obtained from molecular tests, RT-PCR results, from different hospitals. More explanations are added in line number (version with all markups) 150-156; 202-209; 232-233.

3-The authors must add more details and information about the limitations of the methodology which they have reached in the manuscript where there are multiple studies represent how the same methodology appears its limitations because this is a significant point.

Response: More detailed information added in line number (version with all markups) 569-578

4-In the figure 6, the authors must retype the actual units of the measurement.

Response: Actual units of measurements were added in Figure 2. In Figure 6, normalized counts were shown.
5-The figure 2, 3 and 4 in addition to the related results of the manuscript need more comparisons in the light of the methodology itself.

Response: More comparison added for -

Figure 2, line number (version with all markups) 372-385; 395-400; 410-417; 424-439.

Figure 3, line number (version with all markups) 445-468

Figure 4, line number (version with all markups) 487-497

6-The availability of the methodology which the authors have used in the manuscript must be expanded to other problems.

Response: The XGBoost models selected from internal evaluation were expanded to external evaluation, line number (version with all markups) 586-590; and blind predictions line number (601-610)
7-In the conclusion section of the manuscript, the authors must add more details about the future improvements of the suggested methodology in the light of the methodology of the manuscript and about its availability to other measuring problems.

Response: Suggestions incorporated. Line number (Version with all markups) 664-667
8-There are some typographical or grammatical mistakes found in the text of the manuscript, so, the authors must recheck all of those mistakes over all parts of the manuscript.

Response: The typographical or grammatical mistakes were rechecked manually and with Grammarly (https://app.grammarly.com/apps).
9-There are some recent literature studies about the transmission of COVID-19 in Brazil using of the machine learning techniques which is doi:10.1515/em-2021-0029, and doi:10.1515/em-2020-0036, and doi:10.1515/em-2022-0108, and the authors must discuss those literature studies as examples of those studies.

Response: References included, line number (version with all markups) 104-106, reference numbers; - 27 and 28. The third reference suggested was not aligned to the current work – not included.

10-The statistical analyses of the methodology for the rat is pure, it is preferable mention the positive and the negative point for each case.

Response: Suggestions included, line number (version with all markups) 58-79

11-There are some indicators of the used methodology are not included where there are multiple effects of those indicators.

Response: Authors assumed that the reviewer mentioned indicators as hematological parameters. Those discussions were incorporated in line number (version with all markups) 91-100; 117-121.

12-In the table 1 of the manuscript, there is missing discussions of some significant details, and it must be taken into account.

Response: Discussions added, line number (version with all markups) 166-170; 211-216

13-the table 2 of the manuscript needs more discussion because this table is a key-role of the results of the manuscript.

Response: Discussion added, line number (version with all markups) 528-555

14-The availability of the methodology procedures of the manuscript which the authors have employed must be expanded by adding more information and details.

Response: More information and references were added, reference numbers 42-48.
15-There are some symbols or abbreviations used in the text of the manuscript without any definition, so the authors must define each symbol or abbreviation even if the symbol is very well known.

Response: Abbreviations added at the end, line number (version with all markups) 682:701
16-The authors must show the negative effects of the ML model.

Response: Suggestions incorporated. Line number (version with all markups) 659-664
17-The authors must retype all of the equations of the manuscript in a more effective way.

Response: Suggestions incorporated. Line number (version with all markups) 349-351, Eq 1-3.

Reviewer #2: Introduction

Early detection of severely infected COVID-19 patients, ICU admission, and comprehensive information from previous studies on mortality estimation are important for understanding the importance of the subject. This information to be added will increase the validity and reliability of the study. In addition, this study draws attention to some biomarkers in the diagnosis and determination of the severity of COVID-19. Your reason for choosing these features will be better understood if you consider the explanations I have suggested below. Pay attention to these suggestions.
First of all, in the introduction of this study, a paragraph should be written about the importance of routine blood values, which are effective in the diagnosis, prognosis and mortality of COVID-19.

Response: Suggestions incorporated line number (version with all markups) 83-100
1) There is important information in the following articles that point to the role and importance of laboratory markers in the diagnosis, prognosis and mortality of COVID-19. I suggest citing the key findings of these articles.
-What Is the Impact and Efficacy of Routine Immunological, Biochemical and Hematological Biomarkers as Predictors of COVID-19 Mortality? Int. Immunopharmacol. 2022, 105, 108542. <https://doi.org/10.1016/j.intimp.2022.108542>.

Response: Reference incorporated, reference no., 13
-The Effectiveness of Blood Routine Parameters and Some Biomarkers as a Potential Diagnostic Tool in the Diagnosis and Prognosis of Covid-19 Disease. Int. Immunopharmacol. 2021, 98, 107838. <https://doi.org/10.1016/j.intimp.2021.107838>.

Response: Reference incorporated, reference no., 19
-Forecasting of Oxidant/Antioxidant levels of COVID-19 patients by using Expert models with biomarkers used in the Diagnosis/Prognosis of COVID-19. Int. Immunopharmacol., 2021; 100, 108127. <https://doi.org/10.1016/j.intimp.2021.108127>.

Response: Reference incorporated, reference no., 20
2) There is important information in the following articles emphasizing the changes in routine blood values and some demographic characteristics in the deterioration of immune responses and the increase in the severity and mortality of the disease in COVID-19. I suggest citing the key findings of these articles.
-COVID-19 Is More Dangerous for Older People and Its Severity Is Increasing: A Case-Control Study. Med. Gas Res. 2022, 12, 51–54. <https://doi.org/> 10.4103/2045-9912.325992.

Response: Reference incorporated, reference no., 17
-How Do Routine Laboratory Tests Change in Coronavirus Disease 2019? Scand. J. Clin. Lab. Investig. 2021, 81, 24–33. <https://doi.org/10.1080/00365513.2020.1855470>.

Response: Reference incorporated, reference no., 40
-Oxyhemoglobin Dissociation Curve in COVID-19 Patients. Meandros Med Dent J 2023;24(1):58-64. <https://doi.org/10.4274/meandros.galenos.2023.87049>.

Response: Reference incorporated, reference no. 41
-Prediction of Diagnosis and Prognosis of COVID-19 Disease by Blood Gas Parameters Using Decision Trees Machine Learning Model: A Retrospective Observational Study. Med. Gas Res. 2022, 12, 60–66. <https://doi.org/10.4103/2045-9912.326002>.

Response: Reference incorporated, reference no., 29
3) The following articles describe the efficacy of important biomarkers as a potential predictor of diagnosis, prognosis and mortality of COVID-19. I propose to refer to the main findings of these articles.
-Diagnosis and Prognosis of COVID-19 Disease Using Routine Blood Values and LogNNet Neural Network. Sensors 2022, 22, 4820. <https://doi.org/10.3390/s22134820>.

Response: Reference incorporated, reference no., 30
-Automatic Detection of Severely and Mildly Infected COVID-19 Patients with Supervised Machine Learning Models. IRBM 2022, 1, 1–12. <https://doi.org/10.1016/j.irbm.2022.05.006>.

Response: Reference incorporated, reference no., 31
-Detection of Risk Predictors of COVID-19 Mortality with Classifier Machine Learning Models Operated with Routine Laboratory Biomarkers. Appl. Sci. 2022, 12, 12180. <https://doi.org/10.3390/app122312180>.

Response: Reference incorporated, reference no., 32
-Machine Learning Sensors for Diagnosis of COVID-19 Disease Using Routine Blood Values for Internet of Things Application. Sensors 2022, 22, 7886. <https://doi.org/10.3390/s22207886>.

Response: Reference incorporated, reference no., 33
-Effect of ferritin, INR, and D-dimer immunological parameters levels as predictors of COVID-19 mortality: A strong prediction with the decision trees. Heliyon, e14015. <https://doi.org/10.1016/j.heliyon.2023.e14015>.

Response: Reference incorporated, reference no., 34
4) Findings should not be mentioned in the introduction. The sentences that are suitable for the hypothesis and the purpose of the article are sufficient.
Response: Suggestions incorporated, line number (version with all markups) 107-111
Material and Method
-Information about patient exclusion criteria and demographic characteristics should be given.

Response: Suggestions incorporated, line number (version with all markups) 152-154; 202-209; 241-243
-Which features are taken should be stated in units of these features. This is important for clinicians.

Response: Unit were added in Figure 2
-I suggest that the explanations of machine learning algorithms should be given as sub-headings. In addition, literature should be given for these explanations.

Response: Sub-headings added for individual machine learning algorithms in method section
-Is the feature selection model used for machine learning models? should be specified.

Response: Feature selection was revisited. In the revised version feature selection was based on the common parameters across different countries, line number: 431-442
- It should be stated what the performance metrics are used for (for example, for diagnostic performance).
Response: Suggestion incorporated, line number 341-345; 360-361
Results
- 95% confidence limits of performance metrics should be given.

Response: Suggestion incorporated, line number 350-353 (Eq. 4), Table 2 and Table 3.
-There is a big difference between sensitivity and specificity results in model performance results. This indicates the imbalance of the data set. Explain what was done to balance and optimize the dataset.

Response: XGBoost, being a ternary classifier, can handle data imbalance on its own. For other models, data imputation was done.

There is a possibility to improve poor sensitivity and specificity by using the training and test dataset from the same analytical instrumentation (line number:664-665). However, implementation of the same is beyond the scope of the study.
- It will be more beautiful if the figures are colored (especially figure 4). In addition, the resolution of the figure should be increased.
Response: All the figures are now in colour
Dıscussıon and Conclusion
- I could not see a comparison of the important findings in this study with the literature. Discussion on this should be added. You can also use the above-mentioned articles for this.

Response: Suggestions incorporated – “Results and Discussions” – subsection “*Comparison of internal performances of the XGBoost model with published reports”,* line number, 525-555
-Which features were most effective in the diagnosis of the disease? these should be specified.

Response: Suggestion incorporated – “Conclusions” – line number: 652-657
- The prominent findings of the article should be reported in articles.

Response: Suggestion incorporated – “Conclusions” – line number: 652-671

Reviewer #3: The external validity of machine learning-based prediction scores from hematological parameters of COVID-19: A study using hospital records from Brazil, Italy, and Western Europe

The manuscript proposes an interesting investigation on the performance of machine learning techniques to predict scores from hematological parameters of COVID-19. The study also compares prediction performance on data from different countries. The paper is well structured and discussed. Results are properly presented and explained. Here are some questions/recommendations to improve its quality.
• In line 219, authors state that an imputation process was required for ML models other than XGBoost. Was any statistical test conducted to ensure that the imputed information did not change the underlying structure of the data?

Response: Chi-squared test was performed – “Method” section, line number 330-336.
• What is the proportion between training and testing sets? How was that splitting performed? Could different proportion between training and testing affect the performance of the classifier?

Response: Proportion of training and testing sets was 90:10 (line no. 308-309). A 10-fold cross-validation was performed, which showed little effect on the classifier's performance with different sets of training and testing data, line number 485-487 and Table S4.

• In line 254 it is mentioned that hematological features were selected based on the Pearson correlation coefficients between the features and the SARS-COV-2 results (positive or negative). Any concern on the correlation among the features themselves? Several classifiers tend to have their performance reduced when dealing with highly correlated features. Please comment on it. Also, were these retained features meaningful from a practical perspective?

Response: Pearson correlation coefficient was replaced by point biserial coefficients. In the revised version of the manuscript, the hematological features were not selected based on correlation coefficients only. Selection of the features were described in details in Results and Discussion” section – sub-heading – “*Correlation between features and SARS-COV-2 results in different clinical datasets” –* line number: 370-442
• How were the cutting limits for the Pearson correlation defined?

Response: Not relevant in this revised version.
• I would recommend a minor comment on the computational processing time required by each technique (if possible), since more complex techniques tend to be very time-consuming.

Response: Elapsed time for different models were reported in Table S6; line number: 500-502
• Results on Table 3 a) are substantially smaller than previous ones. Please provide further explanation on such lower performance metrics.

Response: Suggestion incorporated in “Conclusion” – line number 659-664

Reviewer #4: The paper presents an application of Machine Learning methodologies to predict positivity to SARS-CoV-2 based on blood exams results.
I appreciated the attempt to include a diverse set of countries and contexts. Nonetheless, I believe this work has significant limitations.

Major comments

1) I find it puzzling why correlations are used a priori to select features. Models like XGBoost work well also with a large number of features and are capable of automatically selecting the most relevant ones or identifying meaningful combinations. Relying solely on linear pair-wise correlation coefficients to exclude features based on their relationship with the outcome might hinder the discovery of valuable interaction patterns. These interactions may involve features that lack significant predictive power individually but demonstrate strong predictive ability when considered in combination with other features
Response: As suggested by the reviewer, correlations, used a priori to select features – are dropped. Details of feature selection, in this revised version, was added in the “Results and Discussions” section – sub-heading – “*Correlation between features and SARS-COV-2 results in different clinical datasets”*, line number: 370-440
2) The Pearson correlation coefficient is not the most appropriate measure to compute the correlation between a binary variable and a continuous or categorical variable. Indeed, the Pearson correlation coefficient is specifically designed to measure the linear relationship between two continuous variables. More suitable approaches should be considered instead (point-biserial correlation or biserial correlation, other non parametric tests).
Response: Suggestion was incorporated. Pearson correlation coefficient was replaced by point biserial correlation. Results were shown in Figure 2 and in the “Results and Discussions” section – sub-heading – “*Correlation between features and SARS-COV-2 results in different clinical datasets”*, line number: 370-440
3) The motivation of the paper appears to be insufficiently convincing. While the paper emphasises the advantages of haematological analysis, it overlooks significant limitations. For instance, the paper fails to acknowledge that RT-PCR, despite being more expensive, allows for simpler sample collection through nasal or oral swabs, unlike haematological analysis, which requires a blood sample.

Response: Suggestions incorporates. RT-PCR method was acknowledged in the “Introduction” section – line number: 58-64

Additionally, the paper neglects to address rapid tests, a relevant aspect that warrants discussion. Including a comprehensive analysis of different testing methods and their respective pros and cons would significantly enhance the paper's credibility and provide a more balanced perspective.
Response: Suggestions incorporated. Rapid tests were acknowledged in the “Introduction” section – line number: 68-79
4) Regarding the last point, the current framing appears to have a significant issue. The majority of the findings in the presentation revolve around the model's performance, which raises concerns. While the model's performance may be highlighted, it fails to demonstrate the practical advantages of using such a model. There seem to be limited benefits to adopting this model when individuals could obtain similar, if not more accurate, results using a rapid test or PCR without the need for blood collection.

Additionally, the paper indicates that the model's performance dramatically declines when trained on a different population. The information presented in table 3A and 3B suggests that the model behaves similarly to a random classifier in such cases. This is a critical limitation as it contradicts the emphasis on the model's performance.

Response: The limitations and advantages were discussed in the “Conclusion” section – line number: 659-671

It becomes challenging to justify the practical application of this model when its effectiveness diminishes significantly when applied to different populations. See for example the last sentence of the abstract “Thus, current models can be applied at other demographic locations.” I find it very hard to support this statement looking at the results from Table 3.

Response: As suggested, the confusion appeared at the end of the abstract was clarified – line number 37-40

Minor comments
1) The description of the Web application is not very useful and do not add much to the paper, therefore I believe it should be shortened significantly and moved eventually in the Supplementary Information

Response: Suggestion incorporated. The description of the Web application was moved to Supplementary information; line number 640-641

2) The dataset description is quite hard to follow, why so many variations of the same dataset are produced? I’d suggest presenting the results for selected datasets in the main, and move the rest to the supplementary information. Also, there are many arbitrary decisions in the dataset creation part that need justifications (examples are the threshold of null values used to drop entries which are not even consistent across dataset, in some cases it is 90%, in others 66%, why?)

Response: As suggested, we have reduced the number of sub-datasets, those were only relevant to our current study. 90% threshold was used only for dataset 1, which was already small in size. However, for other datasets the threshold was consistently used as 66%; line number 163-164; 212-215.
3) References to ML models and packages used should be included

Response: References to ML models and packages were incorporated in the method section (Reference no., 42, 43, 44, 45, 46, 47 and 48.

4) I could not find details on model training criteria (cross-validation, parameter selection, etc)Response: Suggestions incorporated in the “Method” section – line number 307-309 and 485-487; and Table S4
