# Supplementary material for: The external validity of machine learning-based prediction scores from hematological parameters of COVID-19: A study using hospital records from Brazil, Italy, and Western Europe
Source: PLoS One. 2025 Feb 4;20(2):e0316467. doi: 10.1371/journal.pone.0316467 (PMC11793750; doi:10.1371/journal.pone.0316467)
Supplement: Attachment — Submitted filename: response_to_reviewers31032024.docx [file pone.0316467.s003.docx]

 Response to the reviewers

Reviewer #5: The research is significant as it addresses the need for rapid, accurate, and cost-effective COVID-19 detection methods, especially in large-scale and low-income populations.
However, the manuscript, even in its revised version, still has numerous flaws that need to be addressed to allow for its publication.
1) The authors claim that used an innovative approach combining machine learning with hematological parameters for COVID-19 prediction, but they do not consider previous studies that have already been published on this matter as published in [1].

Ans: The above-mentioned reference (reference 35) was cited in the text (“..all-markups” file: line 105). The text was modified accordingly my removing the statements including innovative approaches (“..all-markups” file:lines 40-41)..

Again, authors claim that the novelty lies in the external validation of the ML models across different populations and countries, which has not been explored in previous studies. Unfortunately, they do not consider [2], a study published two years ago with different dataset collected across 3 different continents.
Ans: The above-mentioned reference (reference 36) was extensively cited in the text (“..all-markups” file: lines 105, 149, 350, 389, 609, 614, 618, 625, 632 and 644). The statement “the novelty lies in the external validation of the ML models across different populations and countries, which has not been explored in previous studies” was removed.

2) The diminished effectiveness of the model when trained with data from one population and applied to another raises concerns about its wider applicability.

Ans: “The diminished effectiveness of the model when trained with data from one population and applied to another” has been illustrated in detail, citing evidence from reference 36 and present results (“..all-markups” file:lines 144-149, lines 617-622, lines 629-638)

The authors mention that “the combination of CBC parameters varies with ethnicity” (line 98), yet they provide no further elaboration or references to support this assertion. It's crucial, in my view, for the authors to delve deeper into this premise, as it underpins the main argument against the reliability of tools utilizing laboratory data across diverse datasets. I highly recommend a thorough review of reference [3].
Ans: As suggested by the reviewer, we delved deeper into the point - “the combination of CBC parameters varies with ethnicity”. Elaboration and references are added in the text (“..all-markups” file:, lines 109-134).
3) Another significant limitation of this paper is the complete absence of details about the methodologies employed for the laboratory tests. While it's acknowledged that hematological instruments tend to be more standardized compared to clinical chemistry measurements, it is essential to specify the instruments used for the Complete Blood Count (CBC). This is necessary to identify potential discrepancies in results (such as platelet counts) that may arise from different technologies. This factor is another key aspect affecting the reliability of tools developed using machine learning, as elucidated in [4].

Ans: As suggested by the reviewer, the information on the instruments (hematology analyzer) is mentioned in the text “..all-markups” file:line:192; lines 219-220; line:229-230. For datasets 1 and 3, those were not available. Only available for dataset 2.

4) Considering the three major flaws of the work, I prefer not to delve into the details of the manuscript. However, I would like to point out that there are also many small defects to be fixed, such as typographical and grammatical errors noted, indicating a need for careful proofreading. There are many acronyms that are not explained, or they are explained but not the first time they appear in the text. Figure 2 A does not include the units of measurement.

Ans: Units were not available for the dataset 1, based on which Figure 2A was generated.
References

1. Campagner A, et al. External validation of Machine Learning models for COVID-19 detection based on Complete Blood Count. Health Inf Sci Syst. 2021 Oct 23;9(1):37. doi: 10.1007/s13755-021-00167-3.
2. Cabitza F, et al. The importance of being external. methodological insights for the external validation of machine learning models in medicine. Comput Methods Programs Biomed. 2021 Sep;208:106288. doi: 10.1016/j.cmpb.2021.106288.
3. Carobene A, et al. How is test laboratory data used and characterised by machine learning models? A systematic review of diagnostic and prognostic models developed for COVID-19 patients using only laboratory data. Clin Chem Lab Med. 2022 May 5;60(12):1887-1901. doi: 10.1515/cclm-2022-0182.
4. Campagner A, et al. Everything is varied: The surprising impact of instantial variation on ML reliability. Applied Soft Computing Volume 146 October 2023 Article number 110644 doi:10.1016/j.asoc.2023.110644

Reviewer #6: The study addresses the critical issue of COVID-19 diagnosis, leveraging machine learning (ML) techniques to analyze hematological parameters. Its novelty lies in assessing the external validity of these ML-based predictions across diverse populations from Brazil, Italy, and Western Europe, using a significant sample size of 195,554. However, as I will argue later, this is not an original idea and the authors missed to consider a relevant previous contribution t this matter.

But to begin, I want to point out a sentence that I found puzzling: the authors write that "For the first time, we showed that the models trained and tested on the same population produce significant accuracy." This statement is the most obvious thing that can be said in the context of validating machine learning models, and I think it is either a typo or a sentence that the authors should rephrase or delete (more importantly, what do they mean by "for the first time"?).

Ans: The statement has been removed and rephrased, as suggested by the reviewer (“..all-markups” file: lines 40-45)
Second, I noticed that the authors did not consider perhaps the most important work conducting external validation of models that similarly try to diagnose covid-19 from routine blood chemistry data.

Cabitza, F., Campagner, A., Soares, F., de Guadiana-Romualdo, L. G., Challa, F., Sulejmani, A., ... & Carobene, A. (2021). The importance of being external. methodological insights for the external validation of machine learning models in medicine. Computer Methods and Programs in Biomedicine, 208, 106288.
(<https://doi-org.unimib.idm.oclc.org/10.1016/j.cmpb.2021.106288>)

Ans: The above-mentioned reference (reference 36) was extensively cited in the text (“..all-markups” file: lines 105, 149, 350, 389, 609, 614, 618, 625, 632 and 644).
The authors should therefore.
1) consider the degree to which their features overlap with the models presented in that work and, if there is any significant difference, justify that their model is more parsimonious or uses features that cost less to detect.

Ans: Detail discussion was added (“..all-markups” file: lines 623-654)

2) Consider whether their best model can outperform the one presented in that paper, which is already a few years old. If I am not mistaken, their model is also accessible with a free online application, accessible here: <https://covid19bloodtests.pythonanywhere.com/>.

Ans: Yes, one of the models (trained on dataset from Italy and tested on dataset of Brazil) has outperformed the one presented in that paper (“..all-markups” file: line 642-646)

3) In conducting step 3 they should plot their ROC curve and compare it with that of other authors. If their best model's curve intersects their best model's curve at some point, then they should not compare AUC scores to determine which is the best model. Instead, the comparison should be conducted by comparing the balanced accuracy, accuracy and F1 scores with the rspective confidence intervals.

Ans: balanced accuracy, accuracy and F2 scores were reported (Table 3).

4) I strongly recommend plotting even for their models (or at least their best model) an External Performance Diagram (accessible here: <https://qualiml.pythonanywhere.com/?tool=pills-plt2>).

Ans: External performance diagram was plotted (Figure 6).

The similarity between (internal) and external training datasets can be calculated with this tool: <https://psicorrespondence.pythonanywhere.com/>. I am citing work that is all clearly stated in the work cited above.
Ans: The similarity between internal and external datasets were computed using Kolmogorov–Smirnov (KS) test (“..all-markups” file: line: 353-359). The online similarity tool indicated by the referee had some technical glitch; hence, we used the alternate method.

I noticed that I was invited to review a second revision. I noticed that the reviewers involved in the first round raised three important issues, which I also share.
First, I also need a clarification on the handling of dataset imbalances and the rationale behind dataset variations.

Ans: Imbalance in dataset was handled during external data validation by computing “Balanced Accuracy” and F2-score (“..all-markups” file: Table 3, line 360-380, 570, 592,

Second, I also believe that the manuscript needs a more robust justification for the practical applicability of their model. Utility, in terms of Standardized Net Benefit could be evaluated. Third, last but not the least, the previous reviewers noticed that the study did not provide a comprehensive comparison with existing literature.

Ans: A new section has been added by comparing the results with the existing literature (“..all-markups” file: lines 623-654)

Since I also believe that the most important study to date on their same field has been neglected, I also believe that the authors should improve their work before this can be considered acceptable for publication for an important venue as Plos One. I would add that if the authors fail to demonstrate that their model is better than the one presented in Cabitza et al. for example, in balanced accuracy, F1 score, utility or robustness (as might be inferred from the External Performance diagram that I requested them to report in the manuscript) then their work should *not* be published, because it does not represent an innovation in the field compared to the available SOTA. In that cae, however, there might also be some value in testing the best SOTA with the datasets the authors have collected to check its practical robustness.

Ans: I humbly ascertain that Balanced accuracy, F2 score were superior in some of the models presented in this study compared to the existing literature (we were unable to compute the standardized net benefit due to the technical glitch of the online tool to compute “External Performance Diagram”, suggested by the reviewer). SOTA is mentioned in the abstract and in the text (“..all-markups” file: lines 42-45 and 623-654)
