# Supplementary material for: The external validity of machine learning-based prediction scores from hematological parameters of COVID-19: A study using hospital records from Brazil, Italy, and Western Europe
Source: PLoS One. 2025 Feb 4;20(2):e0316467. doi: 10.1371/journal.pone.0316467 (PMC11793750; doi:10.1371/journal.pone.0316467)
Supplement: Attachment — Submitted filename: Response to the Reviewers Comments 13082024.docx [file pone.0316467.s004.docx]

Review Comments to the Author

Please use the space provided to explain your answers to the questions above. You may also include additional comments for the author, including concerns about dual publication, research ethics, or publication ethics. (Please upload your review as an attachment if it exceeds 20,000 characters)

Reviewer #5: The manuscript has undoubtedly improved compared with the previous version. However, I am always surprised when authors only partially follow the suggestions of reviewers. On page 29, the authors stated:
"To note, machine learning (ML) models have inherent limitations, including dependency on datasets, size of the datasets, ethnic variabilities, phenotypic variabilities, and analytical instrumentation for clinical chemistry tests, etc."

Nevertheless, they failed to provide the necessary references as suggested in my previous revision. There is extensive literature on ML models developed using laboratory data that fails to acknowledge these data. As a result, most of these algorithms are not adopted in clinical practice. The specifics of the instrument, unit, and method are not "superfluous" information; without these details, the algorithm cannot be reproducibly applied in other clinical settings. This significant limitation of the study should be clearly stated.

Response: The reference is added on i) page 23; line 539 and page 30; line 712-714.

Reviewer #6: I thank the authors for providing me with a point-by-point response letter. This is a very important thing that I also expect for the next response. I am happy to state that I believe the authors are on the right track. However, they have not yet reached a completely acceptable manuscript, for the reasons I'll share in what follows
While I am very pleased that the authors have considered addressing robustness, I feel that my comment about the utility has been almost entirely disregarded. It is necessary for the authors to calculate the utility of the system, which is even more important since the authors insist on talking about it without measuring it ("The results promise the utility of these models when trained and tested on the same populations.").

Response: The utility was estimated using standardized net benefit. The standardized net benefit equation was adopted from a cross-reference (equation 3 of Riley et.al. Statistics in Medicine, 2021, ug 30; 40(19):4230-51) of the reference, suggested by the reviewer; reference - 36 in this manuscript. As the online tool, suggested by the reviewer, is currently not working we could not replot the robustness diagram. The standardized net benefit values at different threshold values were reported in Table 3.

Personally, I am not aware of any other online tool for calculating the utility of a classification system other than the one available at this address: <https://modelutility.pythonanywhere.com/>. However, I think that even the tool used by the authors to calculate robustness should give an indication of the ECI or Brier Score ( which I find less understandable than the ECI). Also in regards to the AUC, I prefer the balanced accuracy score and I think the authors should report that in the robustness figure and not the AUC unless they convince me otherwise.

Response: AUC and Brier scores were depicted against the similarity values in external performance diagram (Figure 6 and page 27; line 636). Although, the reviewer has suggested to report balanced accuracy score instead of AUC in the robustness figure, the online tool (<https://modelutility.pythonanywhere.com>) only provides AUC score and not the balanced accuracy, in the figure. To note, the tool is currently (as of on 8^th^ August 2024) not working. Hence, we could not replot the figure. The advantage of balanced accuracy over AUC score was discussed (page 23; line 551-553). It is worthy to mention that we have not used any online tools. Instead, we used the equations given in several references and have written codes on our own to deduce the values.

Moreover, since the comparison with the State of the Art systems (SOTA) is critical, I am pleased that the authors show that one of their models is indeed better than the existing SOTA, but this noteworthy result should be shown in the robustness figure. SOTA has been shared on the Zenodo platform and is available online to test on authors' cases.

Response: We cannot show the robustness figure as the online tool suggested by the reviewer was not working, currently. However, we tried our best to consolidate the results of utility (in terms of standardized net benefit) in Table 3 and in the text – (page 16: line 361 – line 368); (page 23: line 553 – page 24: line 562); (page 25:line 584-page 26:line 591); (page 27:642 – page 28:651)

Since, as I already "disclosed" to the authors that I was enrolled as a reviewer during the second round to replace a reviewer who was no longer available, I want to make my own a comment that I feel was not adequately addressed by the authors regarding the absence of detailed information about the methodologies and instruments used for laboratory tests, particularly in the context of datasets and the corresponding units of measurement, In my opinion, the solution provided by the authors is still incomplete. However it is critical to underscore the importance of including comprehensive details on the instruments and units used across all datasets, not limited to a subset. The authors' reply addresses the instrument used only for dataset 2 and omits relevant information for datasets 1 and 3. This omission significantly impairs the scientific reliability and reproducibility of the results, which is necessary for any valuable research, but even crucial in studies involving machine learning algorithms. Indeed, the absence of such details can lead to inconsistent application and interpretation of the developed models, as laboratory measurements can vary significantly with different instruments and methodologies. Without standardization or at least a clear acknowledgment and documentation of the differences, any machine learning model developed from this data is at a risk of failing to generalize, thus limiting its practical utility and adoption in clinical settings. As noted in the literature, the lack of reproducibility and methodological transparency is a common reason for the limited real-world applicability of many research findings in the machine learning domain. In particular, the inability to verify the consistency of units and measurement techniques across datasets compromises the integrity of the analysis. I recommend that this limitation be clearly acknowledged in the manuscript to provide readers with a comprehensive understanding of the potential variability and implications for the study's outcomes.

Response: The limitation was clearly acknowledged in page 29; line 712-714, with proper reference. The reference was also added to page 23; line 539.

Thus, all things considered, once authors will have addressed the above issue, as well as the utility issue, and improved the robustness diagram (which I really liked!) I believe the manuscript can be accepted for publication. Thank you for your comprehension and collaboration.
