# Supplementary material for: The external validity of machine learning-based prediction scores from hematological parameters of COVID-19: A study using hospital records from Brazil, Italy, and Western Europe
Source: PLoS One. 2025 Feb 4;20(2):e0316467. doi: 10.1371/journal.pone.0316467 (PMC11793750; doi:10.1371/journal.pone.0316467)
Supplement: Attachment — Submitted filename: ResponseToReviewersComments02102024.docx [file pone.0316467.s005.docx]

Review Comments to the Author

Please use the space provided to explain your answers to the questions above. You may also include additional comments for the author, including concerns about dual publication, research ethics, or publication ethics. (Please upload your review as an attachment if it exceeds 20,000 characters)

Reviewer #5: The authors have made considerable efforts to revise the manuscript, and I acknowledge the improvements that have been made. However, I remain concerned about the lack of novelty in this work, which was a primary point of feedback in earlier reviews. The extensive literature in this field has already covered much of what is presented here, and this diminishes the impact of the current study.

Regarding the suggestion to include the review, the intention was not merely to add a citation but to encourage the authors to consider the issue of "noise" in laboratory data—a factor often overlooked, which leads to the poor performance and limited adoption of these tools in practice. This aspect remains unaddressed.

The manuscript would have benefited from a more in-depth exploration of the "noise" in laboratory data (see <https://doi.org/10.1016/j.asoc.2023.110644>). Including such considerations would have added a layer of depth and relevance to the study, making it more compelling.

While the paper may be suitable for publication, it is unfortunate that, despite the detailed point-by-point revisions received, the authors have missed the opportunity to make it truly unique and of significant novelty in the field.

**Response: The reviewer’s comment has been addressed by adding a separate section on “Impact of Instantial Variation (IV) on the external validation datasets:” pages: 27-29; lines: 660-704. Also mentioned in the conclusion, lines: 755-757 (line numbers according to version marked with markup)**

Reviewer #6: The article is much improved, I must give credit to the authors and indeed thank them for considering my suggestions. In my opinion now only the robustness diagram is missing, to compare it with the previous SOTA (which is the article 36). I personally checked that my previous suggestion was not feasible, as written by the authors in their response, but when I checked all the reported tools were working; to be precise, the tool for evaluating model calibration is available at <https://calibrationassessment.pythonanywhere.com/>; the one for the utility is available here: <https://modelutility.pythonanywhere.com/>; the one for calculating similarity here: <https://psicorrespondence.pythonanywhere.com/> and finally the one for the robustness diagram here: <https://qualiml.pythonanywhere.com/>. I have no special contact with the authors of those tools: I just looked in the html code of the page I had reported earlier, namely: <https://mudilab.github.io/dss-quality-assessment/> and extracted the links to the individual tools.
I believe that adding and discussing that diagram and making the codes and datasets available are necessary and sufficient steps to make the work of adequate quality for publication.

**Response: Finally, we were able to execute the online tool with a different browser. Robustness diagram is now added (Figure 6); lines: 599, 605.**
