# Supplementary material for: The external validity of machine learning-based prediction scores from hematological parameters of COVID-19: A study using hospital records from Brazil, Italy, and Western Europe
Source: PLoS One. 2025 Feb 4;20(2):e0316467. doi: 10.1371/journal.pone.0316467 (PMC11793750; doi:10.1371/journal.pone.0316467)
Supplement: Attachment — Submitted filename: response to the reviewers05112024.docx [file pone.0316467.s006.docx]

Please use the space provided to explain your answers to the questions above. You may also include additional comments for the author, including concerns about dual publication, research ethics, or publication ethics. (Please upload your review as an attachment if it exceeds 20,000 characters)

Reviewer #5: I would like to thank the authors for addressing all comments and suggestions thoroughly. The paper is truly of high quality—congratulations!

Reviewer #6: Authors addressed all my requests. However, to enable reproducibility and acknowledge the due credit, they should state, in the caption on Figure 6, the URL (web address) of the tool they used to generate the Robustness Diagram.

Response: The URL (web address) has been added to the caption on Figure 6

Once this has been done, the manuscript can be accepted without further review.
